# Supplementary material for: The causal effect of stressors on antenatal common mental disorders among pregnant mothers in Dabat district Northwest, Ethiopia: A generalized structural equation model approach
Source: PLOS Ment Health. 2025 Mar 21;2(3):e0000260. doi: 10.1371/journal.pmen.0000260 (PMC12798327; doi:10.1371/journal.pmen.0000260)
Supplement: Attachment — Submitted filename: Response to Reviewers.docx [file pmen.0000260.s002.docx]

**Research title: -The Causal Effect of Stressors on Antenatal Common Mental Disorders Among Pregnant Mothers in Dabat District Northwest, Ethiopia: A Generalized Structural Equation Model Approach**

Corresponding Author: - Helina Abebe

**Subject: Response to reviewer and editor comments for the manuscript (ID PMEN-D-24-00327)**

Dear Karli Montague-Cardoso

Executive Editor

PLOS MENTAL HEALTH

Thank you for the opportunity to revise and resubmit our manuscript “The Causal Effect of Stressors on Antenatal Common Mental Disorders Among Pregnant Mothers in Dabat District Northwest, Ethiopia: A Generalized Structural Equation Model Approach.”

We are grateful to the PLOS MENTAL HEALTH Editorial Office for their role in the review process and to the two reviewers for their critical evaluation of our manuscript. We have carefully considered each raised point and made specific changes in response, which have been highlighted using the track change feature. Below, we provide a detailed, point-by-point response to each issue, outlining the changes made in the revised manuscript.

**Editorial request**

1. Comment: please include a rebuttal letter, marked-up manuscript, and clean manuscript with your resubmission

**Response:** We have prepared all the required documents. This rebuttal letter summarizes the changes made in response to the reviewers' comments and is submitted as a separate file. Additionally, we are submitting the following:

- A marked-up version of the manuscript with all changes highlighted (file: "Revised Manuscript with Track Changes").
- A clean version of the revised manuscript without tracked changes (file: "Manuscript").

2. Ensure all figures imply with the journal criteria

**Response:** We have reviewed and prepared all figures following the journal's guidelines. Additionally, we have uploaded the figure files to the Preflight Analysis and Conversion Engine (PACE) digital diagnostic tool to ensure they meet the required resolution, format, and size standards**.**

**Response to Reviewer #1**

1. **- It is better to add more about the global context to the Ethiopian context.**

Response: We sincerely thank the reviewer for their valuable feedback. We have carefully considered their comments and provided detailed responses to each point raised.

Global Context of Antenatal Common Mental Disorders (ACMD):

We have revised the introduction to include a broader perspective on ACMD to address the suggestion of adding a global context. The updated content has been incorporated into the introduction section and can be found on page 2, lone number (63-70)

**Please clarify the statement “prevalence of common mental disorders (CMD) among pregnant mothers that ranged from 12% to 37%,” whether it is local or national data.**

Response: We appreciate the request for clarification regarding the statement, “Prevalence of common mental disorders among pregnant mothers ranged from 12% to 37%.” This range reflects the local data from studies conducted in different regions of Ethiopia: a 12% prevalence was reported in Butajira, while a 37% prevalence was observed in Kersa and Haromaya HDSS. To enhance clarity, we have revised the manuscript accordingly and included this explanation in the introduction section on page (2) and line number (72-75).

2. **It is better to add the rationale for the prevalence of CMD**

Response: - Thank you for your insightful comment. We agree that providing a rationale for the observed variations in the prevalence of Common Mental Disorders (CMD) across different regions of Ethiopia would strengthen the manuscript.

We have revised the manuscript to include this rationale, highlighting the influence of social determinants of health and regional disparities in resource distribution and mental health awareness. The updated content can be found on page 2 and lines 75-79.

3. **Explain why the authors have only included pregnant women in the second and third trimesters since the prevalence of CMD varies across trimesters.**

Response: Thank you for highlighting the need to clarify the rationale for focusing on the second and third trimesters.

Firstly, during the first trimester, many pregnant women experience morning sickness, which includes symptoms such as nausea, vomiting, and fatigue; these physical symptoms often overlap with the somatic manifestations of common mental disorders, such as fatigue, change in appetite, and loss of sleep making it challenging to differentiate between the two. This overlap makes it difficult to distinguish between typical pregnancy discomfort and mental health issues, potentially leading to misinterpretation or misdiagnosis.

During the second and third trimesters, pregnant individuals experience significant hormonal fluctuations that can influence mood and emotional well-being. The increase in hormones such as progesterone and estrogen can lead to heightened emotional sensitivity and vulnerability to mood disorders. Research indicates that these hormone changes can exacerbate pre-existing mental health conditions or contribute to the onset of new ones, making this period critical for assessment. In addition, the second trimester is often characterized by increased awareness and bonding with the fetus, which can trigger complex emotional responses. This period may also bring about anxiety related to impending motherhood, body image changes, and concerns about parenting. By the third trimester, the anticipation of childbirth can lead to heightened anxiety and stress levels, which are crucial times to identify and address common antenatal mental disorders. as they are more likely to surface in a context free from the confounding effects of early pregnancy symptoms.

In summary, these trimesters are characterized by significant emotional and physical changes due to hormonal fluctuations, making it a critical time for understanding their impact on mental health. Additionally, the letter stages of pregnancy are crucial for fetal development; the mother's emotional well-being can profoundly influence fetal outcomes, which our research aims to elucidate during these pivotal months. Finally, many prior studies, including ours, focus on the impact of maternal mental health on perinatal outcomes, which become increasingly relevant as the pregnancy advances. By concentrating our research on the second and third trimesters, we believe we can obtain a clearer picture of the prevalence and impact of common mental disorders during these critical periods of pregnancy.

We have updated the inclusion criteria for the method section of the manuscript on page 5 and lines 128-131.

4. **- How does the ODK software support the data accuracy?**

Response: We appreciate the reviewer's question regarding how ODK (Open Data Kit) software supports data accuracy. ODK is a robust open-source platform designed to enhance the quality and reliability of data collection, particularly in low-resource settings. In the manuscript, we have included detailed explanations on pages 5 and 154-161 to assure the audience that ODK software supports data accuracy and the key ways ODK ensures data accuracy.

5. **The authors should explain how the multicollinearity was handled.**

Response: Thank you for your valuable comment regarding multicollinearity. We appreciate the opportunity to clarify how our analysis addressed it.

To assess multicollinearity among the independent variables in our regression model, we calculated the variance inflation factor (VIF) values. All VIF values were below the commonly accepted threshold of 5, with the highest being 1.10. This indicates negligible multicollinearity among the predictors. Since the VIF values were significantly below the threshold of concern, no further adjustments or measures to address multicollinearity were required. Therefore, the independent variables in our model do not exhibit multicollinearity, which could adversely affect the stability of the interpretation of the regression coefficients. This information has been updated in the analysis section of the manuscript on page 8 and lines 196-200

6. **It would be better to have a row percentage for the comparison in Tables 1 and 2, and something similar would also be shown in Figure 2.**

Response: We thank the reviewer for their valuable feedback. We have revised Tables 1 and 2 to include row percentages alongside the raw counts. As suggested, row percentages are presented clearly to facilitate comparison across categories. We believe this addition improves the clarity and interpretability of the results.

We have carefully evaluated Figure 2's contribution to the manuscript and found that it replicates the information provided in Table 2. Table 2 now includes comprehensive details, including row counts and percentages, and sufficiently highlights the key comparisons. To streamline the presentation of results and avoid unnecessary repetition, we have decided to omit Figure 2 from the manuscript. The updated Tables 1 and 2 are on pages 8 and 11, respectively.

**It would be good if the authors could suggest future research, such as other mediators like economic stability or availability of mental health education, that might want to be considered to clarify antenatal mental health dynamics further.**

Response: Thank you for your insightful suggestion regarding potential mediators that could further clarify antenatal mental health dynamics. We agree that additional factors, such as economic stability and mental health education, should be explored. In the context of our current study, we focused on specific mediators directly related to maternal mental health outcomes, given the scope and objective of our research. However, we acknowledge the importance of broader social determination and mental health education, which could significantly impact antenatal mental health and subsequent outcomes. We have incorporated this suggestion in the revised manuscript, highlighting the need for future research to investigate these mediators. This information has been updated in the discussion section on page 18 and lines 381-386.

7. **Can suggest further works in training healthcare workers and mothers' awareness on coping strategies for mental health.**

Response: Thank you for your thoughtful suggestion regarding the importance of training healthcare workers and raising awareness of coping strategies for mental health. We agree that enhancing the capacity of healthcare providers and empowering mothers with effective coping mechanisms is crucial for addressing antenatal mental health challenges. Integrating capacity-building programs for health education to mothers could significantly improve the identification and management of antenatal mental health disorders. In addition, we have included a statement in the conclusion section of the revised manuscript emphasizing the need for future work in this area on pages 19 and 20, lines 417-423.

**Response for reviewer #2**

1. **If this was a cross-sectional study, what does a ratio of 1:5 mean in the sampling?**

Response: Thank you for pointing out the discrepancy in the description of the sampling ratio. We apologize for the typing error in the manuscript regarding the ratio of exposed to non-exposed subjects. The correct ratio used in the sample size calculation was 1:2, not 1:5, as mistakenly stated.

To clarify, this study was part of a larger project intended to investigate the extent of the impact of perinatal common mental disorders on birth, growth, and infant development outcomes in Dabat HDSS Northwest Ethiopia. We have corrected this information in the manuscript on page 5, lines 139-145.

2. **What were the subjects' modal parity and meant gestational age?**

Response: We appreciate your inquiry regarding the study participants' modal parity and mean gestational age. The modal parity of the participants was “2-4” pregnancies, which accounted for 455 individuals, 52. % of whom were in this category, representing the most significant proportion of the samples. The mean gestational age of the participants was 27 weeks, with a standard deviation of 6.41. we have updated Table 2 to include the category for the number of pregnancies as presented in the revised manuscript. This update has been incorporated in the respective section to represent the data better and enhance interpretability.

3. **Figure 2 is incomplete**

Response: Thank you for your observation. We have addressed this issue by carefully evaluating Figure 2's contribution to the manuscript. We have found that Figure 2 replicates the information provided in Table 2. Table 2 now includes comprehensive details, including row counts and percentages, and sufficiently highlights the key comparisons. To streamline the presentation of results and avoid unnecessary repetition, we have decided to omit Figure 2 from the manuscript. **There are spelling mistakes in the tables. See text for other corrections.**

Response: Thank you for pointing this out. We have carefully reviewed the tables and corrected all spelling mistakes. We have also rechecked the entire manuscript to ensure accuracy and eliminate any remaining errors.
